# Recognizing Catatonia Beyond Hypokinesis: Features Linked to Underdiagnosis and Training Targets

**DOI:** 10.3390/jcm14238382

**Published:** 2025-11-26

**Authors:** Octavia Capatina, Adela Hanga, Sonia Tivadar, Denis Paval, Mihaela Fadgyas Stanculete

**Affiliations:** 1 Department of Neuroscience, Iuliu Hațieganu University of Medicine and Pharmacy, Cluj-Napoca 400347, Romania; 2 County Emergency Hospital, Cluj-Napoca 400347, Romaniativadar.sonia8@gmail.com (S.T.)

**Keywords:** catatonia, underdiagnosis, psychiatric inpatients, clinical signs, diagnostic criteria

## Abstract

**Objective**: To estimate how clinical signs shape whether catatonia is recorded during routine inpatient care and to identify training targets that reduce missed cases. **Methods**: We conducted a retrospective chart review at a tertiary psychiatric hospital (July 2019–July 2024). Of 14,907 admissions, 87 had a formal catatonia diagnosis. We screened discharge summaries with 24 terms; 697 encounters with ≥3 terms underwent independent ICD-11 review by two clinicians (third reviewer as needed), yielding 194 additional encounters meeting criteria. We compared patients with a recorded diagnosis with those meeting criteria without a diagnosis and fitted logistic models for diagnostic assignment. **Results**: At the patient level (first admission), 54 diagnosed and 183 underdiagnosed individuals were included. Hypokinetic signs—negativism, mutism, rigidity, and posturing—were more frequent in diagnosed cases, while grimacing and mannerisms were more frequent among underdiagnosed cases. In adjusted analyses, rigidity and negativism were linked to a higher likelihood of a recorded diagnosis, whereas grimacing signaled missed recognition. Medical complication rates did not differ. The longer unadjusted length of stay in diagnosed patients was not significant after adjustment for age and symptom burden. **Conclusions**: Clinicians tend to identify hypokinetic catatonia and overlook movement-rich presentations. A one-minute ICD-11 screen and documentation prompts for parakinetic/hyperkinetic signs are pragmatic steps for training programs.

## 1. Introduction

Catatonia is a major neuropsychiatric syndrome which is characterized by a compound of motor signs, affective signs, and behavioral signs and can present as hypokinetic, hyperkinetic, or parakinetic [1]. Resembling other common psychiatric syndromes like depressive syndrome or anxious syndrome, catatonia can appear in the context of a primary mental disorder or secondary to medical condition or a psychoactive substance [2,3]. The prevalence of catatonia varies across different medical settings. It is estimated to be approximately 10%, with reported rates ranging from 5% to 25% in acute psychiatric hospitals [4], around 3.8% in intensive care units [5], and about 3.3% in tertiary neurological units [4]. In acute psychiatric inpatients, the prevalence can range from 4% to 43% depending on the specific psychiatric diagnoses: 43% in mood disorder samples, 4% and 15% in schizophrenia patients, and 10.4% in autism spectrum disorders [6]. This variability is believed to be accounted for by the underdiagnosis of catatonia, which was buried under the diagnosis of schizophrenia for the last hundred years and resurrected by the latest diagnostic manuals DSM-5, DSM-5-TR, and ICD-11 [7,8].

Several previous studies have shown that catatonia is often overlooked and that physicians tend to underdiagnose it [9,10,11]. Some key factors leading to underdiagnosing the catatonic syndrome have been discussed by previous research. The misunderstanding of the heterogeneity of catatonia is one of the factors, as many understand catatonia as only a hypokinetic state. The diversity inherent to catatonia makes it hard to be recognized even for trained physicians [12,13]. Another factor is considered to be the multitude of differences across different diagnostic criteria and rating scales. Comparison between diagnostic criteria and rating scales has shown significant discrepancies regarding the items included and also in the definition of the items [10,14]. Only the latest versions of the diagnosis manuals: DSM-5, DSM-5-TR, and the ICD-11 describe catatonia as an independent syndrome, and they also increase the threshold for the diagnosis to a minimum of three criteria and they allow diagnosing catatonia in the context of psychotic disorders, mood disorders, medical conditions, neurodevelopmental disorders and also have an option for unspecified catatonia. Notable differences between the manuals are the inclusion of substance-induced catatonia only in the ICD-11, number of items included in the diagnostic criteria 12 for the DSM and 15 for the ICD. While rigidity, verbigeration, staring, and ambitendency are [2,3] encompassed only by the ICD, echolalia and echopraxia are individual items in the DSM and are merged in the ICD as echophenomena. Increased psychomotor activity is included in both systems of diagnosis, but very different perspectives on the phenomena are described, whereas the DSM provides a single item, agitation, with no definition, the ICD lists and defines three items: agitation, impulsivity and combativeness, mentioning that multiple manifestations of increased psychomotor activity should only be counted as one of the required three criteria for catatonia. There are still ongoing debates in the literature over which items should be included, where there should be a severity ranking, and the duration of their presence in order to diagnose catatonia [1,14].

Regarding the rating scales for catatonia, six different rating scales are available to our knowledge, but the 23-item Bush–Francis Rating Scale (BFCRS) is the most widely used in clinical settings and in research. Despite its ease of administration, good reliability and validity, and free video training modules, several studies reported inaccuracies regarding physicians’ abilities to recognize catatonic features [12,13,15].

Other research concerning the accuracy of the diagnosis of catatonia in different medical settings showed some unsatisfactory results. One retrospective study identified 18 patients who met the criteria for catatonia out of which only two were formally diagnosed [16]. A chart review study evaluating the underdiagnosis of catatonia in the general hospital revealed that for the 133 subjects meeting the DSM-5 criteria for catatonia, only 54 had a documented diagnosis [11]. A prospective cohort study investigated the overlap between delirium and catatonia in critically ill patients, given that traditionally a diagnosis of delirium precludes a diagnosis of catatonia, and showed that one third of the patients had both diagnoses, with rather important consequences for the management [17].

The current literature suggests that the challenge in diagnosing catatonia consists of its variable clinical picture, lack of consensus concerning item definition, item number, and lack of proper training. Catatonia is known to have a good treatment response to lorazepam or ECT, but left untreated due to under-recognition can lead to serious medical complications, like deep vein thrombosis, pulmonary thromboembolism, stasis ulcers, or malnutrition [18,19,20].

The exploration of biomarkers for catatonia has gained momentum in response to the inherent challenges associated with its diagnostic process. The recent literature has increasingly focused on subclinical inflammation due to its association with various psychiatric disorders [21]. Notably, catatonia has been linked to inflammatory processes in multiple studies [22], with low iron levels and high-sensitivity C-reactive protein (HS-CRP) suggested by some researchers as potential biomarkers for this condition [23].

Recent investigations have assessed newer indices derived from hematological parameters, including the Neutrophil-to-Lymphocyte Ratio (NLR), Total Lymphocyte Ratio (TLR), Monocyte-to-Lymphocyte Ratio (MLR), Systemic Inflammation Response Index (SIRI), and Systemic Immune-Inflammation Index (SII), to determine their correlation with catatonia. Among these, the NLR has been found to be significantly elevated in patients with catatonia compared to the general population [24].

The present study was undertaken to identify the frequency of catatonia in a tertiary psychiatric hospital based on a retrospective chart review. The aim was to identify potentially underdiagnosed cases, based on chart notes and to compare the underdiagnosed patients with patients diagnosed with catatonia in terms of socio-demographic characteristics, clinical presentation, and paraclinical characteristics in order to identify factors leading to underdiagnosis. Additionally to exploring potential factors contributing to the underdiagnosis of catatonia, we try to link these factors to training targets which could improve the diagnosis.

## 2. Materials and Methods

We conducted a retrospective chart review of inpatient psychiatric admissions from July 2019 to July 2024 at a tertiary psychiatric hospital.

In order to find the potentially underdiagnosed catatonia patients, we made a list of 24 keywords describing catatonia-related symptoms (Table 1), which we picked from the Bush–Francis Catatonia rating scale, the most widely used scale for catatonia and searched the electronic medical charts of all inpatients from Cluj-Napoca Psychiatric Clinic for the same period of five years.

The presence of three or more catatonia-related symptoms in the discharge papers was used as criteria for identifying underdiagnosed cases. The cases which had the diagnosis of catatonia, or cases where catatonia was mentioned in the patients’ chart, were not reviewed since they were included in the diagnosis group. Given the fact that ICD-10 codes are used in the Romanian medical system, we included in the diagnosis group all charts mentioning catatonia, irrespective of having a formal diagnosis. All chart notes for the cases which included three or more catatonia-related signs and were not included in the diagnosis group were afterwards reviewed for evidence of a catatonia diagnosis, by the use of ICD-11 criteria.

Chart notes for the selected cases were reviewed separately by two of the following clinicians: A.H. and S.T., to see if the description of the case could overlap an episode of catatonia as it is described in ICD-11, and all the included cases were afterwards overviewed by O.C. The charts which were included by only one of the authors were reviewed by M.F.S., D.P., and O.C. to solve the issue and make the final decision by consensus. All catatonia-related signs were attributed to catatonia if there were no better explanation for them.

Socio-demographic, clinical, and paraclinical data were obtained for each patient included in the study. Analyses were performed at the patient level; when multiple admissions occurred, only the first admission was retained.

The study was approved by the Ethics committee of Cluj-Napoca Emergency County Hospital (Approval nr. 31911/7 August 2024).

For the statistical analysis, we used the SPSS version 22.1 package. Descriptive statistics were presented as mean and standard deviation, percentage and frequency of distribution. Kolmogorov–Smirnov and Shapiro–Wilk tests were used to examine the normal distribution of the variables. The two groups, the catatonia group and the underdiagnosed group, were compared using Chi-square and Fisher exact test for categorical variables and Student t-test and Mann–Whitney U-test for continuous variables with Benjamini–Hochberg false discovery rate (FDR) control; tables report *p* and q values, statistical significance at *p* < 0.05 and *q* < 0.05. A stepwise Forward Conditional Binary logistic regression was used to investigate the factors leading to the underdiagnosis of catatonia. A linear regression analysis was used to evaluate differences regarding clinical outcome between the two groups controlling for potential confounders.

## 3. Results

### 3.1. Chart Selection

Between July 2019 and July 2024, a total of 14,907 patients were admitted to the Cluj-Napoca Psychiatric Clinic of the Emergency County Hospital. Among these patients, 87 charts received a formal diagnosis of catatonia and 697 medical records indicated the presence of at least three catatonic symptoms, but did not receive a diagnosis of catatonia. Table 2 delineates the frequency of keywords associated with catatonic signs.

**Figure 1:**
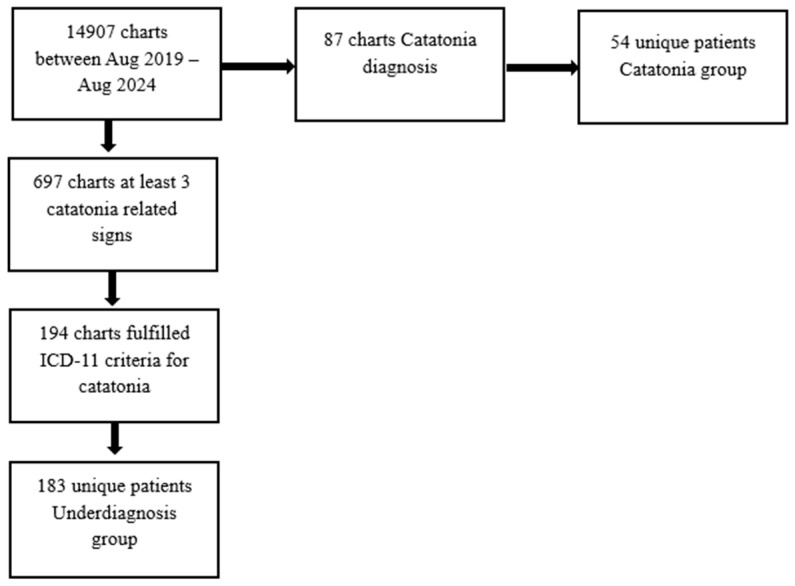
Flowchart of chart selection process.

Following a comprehensive review, it was identified that 194 charts, which had not been previously diagnosed with catatonia, retrospectively fulfilled the ICD-11 criteria for the condition and were thus categorized as the underdiagnosed group (Figure 1). The charts were reviewed by two reviewers and the agreement rate was 84%. We calculated Cohen’s κ = 0.785 with 95% CI (0.743, 0.827) on the double-rated set indicating a substantial agreement; disagreements were resolved by a third reviewer.

For the purposes of this study, two distinct patient groups were defined. The first group, referred to as the catatonia group, comprised individuals who received a formal diagnosis of catatonia. The second group, designated as the underdiagnosis group, included patients who met the ICD-11 criteria for catatonia but did not obtain a formal diagnosis. Out of 87 charts corresponding to patients with a formal diagnosis of catatonia, 54 unique patients were identified; the remaining 33 charts pertained to the same individuals who were admitted on multiple occasions. In the underdiagnosis group, which encompassed 194 charts, 183 individual patients were identified. For those patients who experienced multiple admissions during the study period, data were collected from their first admission.

### 3.2. Comparison of Clinical Characteristics of the Groups

A comparison of the socio-demographic and clinical and paraclinical characteristics of the groups are presented in Table 3.

The univariate analyses conducted between the diagnosed and non-diagnosed groups revealed several significant differences. Notably, patients diagnosed with catatonia were more frequently female (*p* = 0.038) and exhibited a lower likelihood of a diagnosis of affective disorder (*p* = 0.049). However, following the application of the Benjamini–Hochberg procedure for controlling the false discovery rate, these differences no longer retained statistical significance: female gender (*q* = 0.110) and a diagnosis of affective disorder (*q* = 0.126).

Furthermore, individuals diagnosed with catatonia demonstrated a higher prevalence of specific symptoms, including negativism (*p* < 0.000), mutism (*p* < 0.000), rigidity (*p* < 0.000), and posturing (*p* < 0.000) relative to their non-diagnosed counterparts. Conversely, the presence of grimacing (*p* < 0.000) and mannerisms (*p* = 0.011) was significantly associated with the absence of a catatonia diagnosis. Moreover, the lack of echophenomena (*p* = 0.001), catalepsy (*p* < 0.000), and waxy flexibility (*p* < 0.000) were strongly correlated with a non-diagnosis of catatonia. In addition, lower serum iron levels (sideremia) (*p* = 0.015) were associated with the diagnosed group. All these differences retained statistical significance following the application of the Benjamini–Hochberg procedure for controlling the false discovery rate: negativism (*q* < 0.000), mutism (*q* < 0.000), rigidity (*q* < 0.000), posturing (*q* < 0.000), grimacing (*q* < 0.000), mannerisms (*q* = 0.039), echophenomena (*q* = 0.004), catalepsy (*q* < 0.000), and waxy flexibility (*q* < 0.000).

### 3.3. Binary Logistic Regression Predicting the Underdiagnosis of Catatonia

A stepwise forward conditional binary logistic regression analysis was performed to identify predictors associated with the underdiagnosis of catatonia. The predictors entered into the regression model were those that reached statistical significance in the preceding univariate analyses after controlling for the false discovery rate, comparing the diagnosed and non-diagnosed groups. Notably, sideremia was excluded due to incomplete data, as was the total number of ICD-11 criteria for catatonia met, owing to multicollinearity concerns.

The results of this analysis are summarized in Table 4. In the first step of the analysis, Rigidity was entered as a predictor variable. The results indicated that Rigidity significantly decreased the likelihood of catatonia being underdiagnosed, with on odds ratio (OR) of 0.030 (95% CI [0.013, 0.068] *p* < 0.000). In the second step, Grimacing was added to the model. The results revealed that Grimacing, more common among underdiagnosed cases, had an odds ratio of 0.106 (95% CI [0.030, 0.370] *p* < 0.000), indicating lower odds of receiving a catatonia diagnosis. The effect of Rigidity remained significant with an OR of 0.029, 95% CI [0.012, 0.070], *p* < 0.000). In the third step, Negativism was included in the analysis. The results indicated that Negativism was a significant predictor for the diagnosis with an odds ratio of 0.249 (95% CI [0.098, 0.633], *p* = 0.004), Grimacing remained significant (OR of 0.082, 95% CI [0.022, 0.314] *p* < 0.000), and Rigidity maintained its significance (OR of 0.029, 95% CI [0.012, 0.073], *p* < 0.000). Overall, these findings suggest that Rigidity, Grimacing, and Negativism are significant predictors of the outcome.

These findings highlight the complex interplay of clinical features in the diagnostic process of catatonia and underscore the need for heightened awareness of these symptoms to mitigate underdiagnosis.

### 3.4. Clinical Outcome

In the present study, clinical outcomes were assessed through the evaluation of hospitalization duration and the incidence of medical complications associated with catatonia, including deep vein thrombosis, pulmonary thromboembolism, urinary retention, pneumonia, and decubital ulcers. The univariate analysis revealed no significant differences between the groups regarding the prevalence of somatic complications (*p* = 0.425). However, the Mann–Whitney test indicated that the number of days of hospitalization was significantly greater in the catatonia group (*p* = 0.019). Nonetheless, after adjusting for age and the number of catatonia signs through linear regression analysis, this difference did not maintain statistical significance (β = 0.033, *p* = 0.693). These findings suggest that while initial observations indicate prolonged hospitalization for catatonia patients, this association may be confounded by age and clinical presentation, necessitating further investigation to elucidate the underlying factors influencing these outcomes.

## 4. Discussion

The findings of this study underscore the critical issue of underdiagnosis of catatonia within a psychiatric hospital in Cluj-Napoca, Romania, where ICD-10 codes are still widely used. Despite the recognition of catatonia as a distinct syndrome in the latest diagnostic manuals like the DSM-5-TR and ICD-11, our research illuminates the persistent challenges in its identification and diagnosis.

The retrospective analysis revealed that 183 patients exhibited three or more symptoms of catatonia yet did not receive a formal diagnosis. This aligns with the previous literature indicating that catatonia is often overlooked by clinicians, primarily due to misconceptions surrounding its heterogeneous clinical presentations [12,15]. The results corroborate the idea that catatonia is frequently associated with conditions such as schizophrenia [25], leading to a lack of recognition as an independent entity despite exhibiting characteristic signs [26].

Our analysis notably revealed that patients in the diagnosed group were more likely to be female and exhibited pronounced symptoms of negativism, mutism, rigidity, and posturing compared to those in the underdiagnosed cohort. Additionally, the diagnosed group demonstrated a significantly higher number of catatonic symptoms, suggesting that more severe manifestations of the disorder are more readily identified, while the underdiagnosed group may include cases of milder presentation.

These findings underscore the recognition that the hypokinetic form of catatonia is the most frequently acknowledged within clinical settings. Furthermore, the presence of grimacing and mannerisms was significantly associated with the underdiagnosed group, indicating a potential tendency among clinicians to under-recognize the parakinetic and hyperkinetic subtypes of catatonia [27]. This observation aligns with the existing literature, which indicates that hyperkinetic catatonia may be overlooked in children and adolescents [16]. Moreover, previous studies have documented instances where catatonia is misattributed to hypokinetic delirium, particularly in intensive care settings [5], or where parakinetic catatonia is erroneously diagnosed as parkinsonian disorders [17,28]. These patterns highlight the critical need for increased awareness and training regarding the diverse presentations of catatonia to improve diagnostic accuracy.

In our study, the underdiagnosed group exhibited a significant association with affective disorders when compared to the catatonia group, before controlling for the false discovery rate. This finding contradicts the prevailing literature, which indicates that catatonia is most frequently associated with affective disorders [4]. Two potential explanations may account for this discrepancy.

First, in Romania, diagnostic coding continues to rely on the International Classification of Diseases, 10th Revision (ICD-10) [29], which does not recognize catatonia within the context of affective disorders. This limitation may hinder the accurate identification and diagnosis of catatonia among patients presenting with affective symptoms.

Second, there exists a historical perspective within the field that has categorized catatonia as a phenomenon primarily associated with schizophrenia [25]. This classification can persistently influence clinical practice and diagnostic approaches. Alarmingly, some studies investigating contemporary understanding of catatonia suggest that this outdated view continues to prevail among certain clinicians [9,15]. Together, these factors highlight the need for increased awareness and educational efforts to ensure accurate diagnostic practices that reflect the complexities and nuances of catatonia in diverse clinical contexts [12,15].

The logistic regression analysis identified grimacing as a significant predictor of underdiagnosis, underscoring the intricate nature of catatonia’s clinical presentation. Correspondingly, another investigation conducted in a general hospital setting found similar predictors related to symptoms that are frequently attributed to alternative diagnoses [11].

Interestingly, the presence of negativism and rigidity was associated with a decreased likelihood of underdiagnosis, indicating a heightened awareness of these particular symptoms among clinicians. This analysis further supports the notion that clinicians are predominantly familiar with the hypokinetic subtype of catatonia, thereby revealing a potential gap in training and awareness regarding the diverse manifestations of catatonia [12,14]. The difference in presentation likely reflects distinct, though overlapping, underlying neurobiological profiles, contributing to the recognition bias. Recent functional neuroimaging (fMRI, SPECT) studies support the idea that catatonia involves dysregulation in the cortico-striatal–thalamo-cortical (CSTC) loops, cortico-cerebellar, anterior cingulate–medial orbitofrontal, and lateral orbitofrontal networks, particularly affecting motor control, and that different subtypes may involve specific regional abnormalities [30,31]. Classic hypokinetic state may be a product of hyper-inhibition: increased neural activity in premotor areas (e.g., Supplementary Motor Area-SMA and lateral premotor cortex-L-LPM), and decreased GABA-A receptor density in the left sensorimotor cortex), while the less-recognized parakinetic/hyperkinetic state may be a product of disinhibition: functional connectivity abnormalities in the frontoparietal network and decreased activity of the orbitofrontal cortex (OFC). The latter, resembling other common psychiatric emergencies (mania and agitation), is more susceptible to the recognition bias [32]. A significant proportion of catatonia is secondary to medical conditions, particularly those involving inflammation or autoimmunity (e.g., encephalitis, and systemic lupus erythematosus) [33]. Systemic infection or inflammation (high CRP, and Leukocytes) triggers a central nervous system response, leading to ‘sickness behavior’ (e.g., withdrawal, lethargy, and decreased social interaction), and this state of systemic slowing and withdrawal may manifest as the stuporous, hypokinetic features of catatonia [32].

These findings emphasize the necessity for enhanced educational initiatives aimed at improving clinician familiarity with the full spectrum of catatonic presentations to facilitate more accurate diagnoses and timely interventions in affected individuals.

In terms of clinical outcomes, no significant differences were observed regarding the incidence of medical complications between the diagnosed and underdiagnosed groups. However, the number of days spent hospitalized was significantly greater among the catatonia group. This finding correlates with the previous analyses indicating a higher incidence of catatonic symptoms within this group, which typically reflects a more severe clinical presentation. Notably, when controlling for age and the number of catatonia symptoms, the difference in hospitalization duration between the groups lost its statistical significance.

This study reinforces the notion that catatonia continues to be underdiagnosed, with significant implications for patient management and clinical outcomes. The challenges associated with diagnosing catatonia—including prevalent misconceptions regarding its clinical presentation, variability in diagnostic criteria, and reliance on outdated coding systems—contribute to its continued under-recognition within psychiatric settings. The failure to identify this syndrome can lead to adverse medical complications, inappropriate management strategies, and extended hospital stays.

Future initiatives should focus on enhancing clinician education concerning the full spectrum of catatonia’s signs and symptoms, the adoption of contemporary diagnostic criteria universally, and the implementation of standardized screening protocols in psychiatric environments. Increasing awareness and training may effectively reduce rates of underdiagnosis, thereby improving patient outcomes and mitigating the associated risks of untreated catatonia. These findings point to practical training targets: a one-minute ICD-11-anchored intake screen; admission templates that explicitly prompt for grimacing, mannerisms, and echophenomena; brief resident teaching with video examples; and a smart-phrase checklist in the record. Such low-burden steps align recognition with the full spectrum of catatonia.

Several limitations of this study warrant consideration. First, it is important to note that this was a retrospective analysis, which involved reviewing patient charts. Thus, the diagnosis of catatonia in the underdiagnosed group should be interpreted as an assumption rather than a definitive conclusion. Moreover, neither the diagnosed nor the underdiagnosed patients underwent evaluation using validated assessment tools; therefore, our findings rely on the observations and documentation of various clinicians, which introduces potential variability and subjectivity into the data collection process. To overcome these limitations, prospective studies using standardized diagnostic criteria applied in real-time to validate our findings and reduce potential retrospective biases should be conducted. Standardized scales, like the Bush–Francis scale, which also has video training modules, are the best way to reduce variability and subjectivity of data collection, but this of course is not possible in a retrospective design. Future research should prioritize prospective data collection that includes standardized assessments of comorbidity severity, detailed medication histories, and longitudinal data on illness duration to better control for these factors in multivariable analyses. This will improve the interpretability of the results and allow for more robust conclusions regarding the factors associated with the underdiagnosis of catatonia. We acknowledge that without controlling for these factors, the findings are only suggestive and not definitive.

## Figures and Tables

**Table 1:** Keywords defining catatonia-related symptoms.

List of Symptoms from the Bush–Francis Catatonia Scale	
Immobility	Verbigeration
Mutism	Rigidity
Staring	Negativism
Catalepsy	Waxy Flexibility
Posturing	Withdrawal
Grasp Reflex	Combativeness
Grimacing	Impulsivity
Echolalia	Automatic Obedience
Echopraxia	Active resistance
Stereotypy	Autonomic abnormality
Mannerisms	Ambitendency
Perseveration	Stupor

**Table 2:** Frequency of catatonia-related signs.

Keywords	No. of Charts	Keywords	No. of Charts
Immobility	157	Verbigeration	6
Mutism	123	Rigidity	51
Staring	325	Negativism	174
Catalepsy	5	Waxy Flexibility	14
Posturing	10	Withdrawal	505
Grasp Reflex	0	Combativeness	337
Grimacing	10	Impulsivity	344
Echolalia	46	Automatic Obedience	21
Echopraxia	8	Active resistance	0
Stereotypy	355	Autonomic abnormality	0
Mannerisms	339	Ambitendency	82
Perseveration	16	Stupor	20

**Table 3:** Comparison of characteristics of the catatonia group with the underdiagnosed group.

Socio-Demographic Characteristics	Catatonia Group N = 54		Underdiagnosed Group N = 183		*p*	*q*
	Mean	SD	N	SD		
Age	43.3	18.8	44.8	15.9	0.541	0.675
	%	N	%	N		
Female gender	74.1%	40	58.5%	107	0.038	0.110
Clinical characteristics						
Catatonia due to an organic disorder	16.6%	9	12%	22	0.366	0.659
Catatonia due to psychotic disorders	72.2%	39	71%	130	0.789	0.758
Catatonia due to affective disorders	11.1%	6	16.9%	31	0.049	0.126
Past psychiatric history	74.1%	40	77.6%	142	0.590	0.712
Somatic complications	11.1%	6	12%	22	0.425	0.603
**Paraclinical characteristics**						
	Mean	SD	N	SD		
Leucocyte	8.4	2.9	9.9	2.3	0.590	0.691
Neutrophils	5.6	2.6	5.3	2.4	0.503	0.647
Lymphocyte	2.0	0.7	2.0	0.8	0.875	0.875
Monocyte	0.6	0.2	0.5	0.1	0.097	0.227
Thrombocyte	275.3	87.4	250.1	70.0	0.194	0.338
NLR	3.1	1.9	3.1	2.3	0.500	0.673
TLR	153.1	72.8	139.3	70.3	0.228	0.364
MLR	0.3	0.1	0.2	0.1	0.073	0.183
SIRI	2.0	1.6	1.7	2.0	0.108	0.236
SII	867.5	609.3	779.1	617.8	0.177	0.334
CK	328.9	612.7	499.2	2342.2	0.386	0.564
CPR	0.9	1.6	1.1	2.6	0.619	0.723
Sideremia	61.9	33.5	79.8	35.9	0.015	0.048
**ICD 11 criteria for catatonia**						
	N	%	N	%		
Staring	34	62.96%	99	54.07%	0.211	0.358
Ambitendency	15	27.78%	43	23.51%	0.473	0.661
Negativism	39	72.22%	82	44.84%	0.000	0.000
Stupor	34	62.96%	139	75.96%	0.112	0.242
Mutism	42	77.78%	85	46.38%	0.000	0.000
Increased psychomotor activity	22	40.74%	72	39.34%	0.874	0.900
Grimacing	15	27.78%	78	42.62%	0.000	0.000
Mannerisms	13	24.07%	81	44.26%	0.011	0.039
Posturing	23	42.59%	9	4.92%	0.000	0.000
Stereotypy	22	40.74%	82	44.84%	0.754	0.744
Rigidity	41	75.93%	17	9.27%	0.000	0.000
Echophenomena	13	24.07%	13	7.11%	0.001	0.004
Verbigeration	5	9.26%	8	4.38%	0.174	0.319
Waxy flexibility	32	59.26%	14	7.65%	0.000	0.000
Catalepsy	14	25.93%	7	3.83%	0.000	0.000
	Mean	SD	N	SD		
Total ICD 11 criteria met	6.7	2.7	4.1	1.2	0.000	0.000

**Table 4:** Predictors for underdiagnosis catatonia.

	Variable	Adjusted Odds Ratio	95% CI	p	Hosmer and Lemnshaw Test
Step1	Rigidity	0.030	0.013–0.068	0.000	
Step 2	Grimacing	0.106	0.030–0.370	0.000	0.614
	Rigidity	0.029	0.012–0.070	0.000	
Step 3	Negativism	0.249	0.098–0.633	0.004	0.204
	Grimacing	0.082	0.022–0.314	0.000	
	Rigidity	0.029	0.012–0.073	0.000	

## Data Availability

Datasets are available on request; there are no publicly available datasets due to privacy restrictions.

## Abbreviations

The following abbreviations are used in this manuscript:

ICD	International Classification of Diseases
DSM	Diagnostic and Statistical Manual of Mental Disorders
NLR	Neutrophil-to-Lymphocyte Ratio
TLR	Thrombocyte-to-Lymphocyte Ratio
MLR	Monocyte-to-Lymphocyte Ratio
SIRI	Systemic Inflammatory Response Index
SII	Systemic Immune Inflammatory Index
CK	Creatine Kinase
CPR	C-reactive protein
